# Influence of the Enterovirus 71 Vaccine and the COVID-19 Pandemic on Hand, Foot, and Mouth Disease in China Based on Counterfactual Models: Observational Study

**DOI:** 10.2196/63146

**Published:** 2024-12-17

**Authors:** Jia Nie, Tian Huang, Yuhong Sun, Zutong Peng, Wenlong Dong, Jiancheng Chen, Di Zheng, Fuyin Guo, Wenhui Shi, Yuewei Ling, Weijia Zhao, Haijun Yang, Tiejun Shui, Xiangyu Yan

**Affiliations:** 1 School of Disaster and Emergency Medicine, Tianjin University, No. 92, Weijin Road, Nankai District, Tianjin, 300072, China, 86 02287370177307; 2 Institute for Risk Assessment Sciences, Utrecht University, Utrecht, Netherlands; 3 Yunnan Center for Disease Control and Prevention, Yunnan, China; 4 College of Animal Science and Technology, China Agricultural University, Beijing, China; 5 Department of Dermatology and Venereology, the First Affiliated Hospital of Kunming Medical University, Yunnan, China; 6 Xiamen Peiyang BCI & Smart Health Innovation Research Institution, Xiamen, China; 7 People's Hospital of Pu'er, Yunnan, China; 8 People's Hospital of Lincang, Yunnan, China; 9 Lanke Medical Technology Nanjing Research Institution, Nanjing, China; 10 Department of Management Science and Engineering, Stanford University, Stanford, CA, United States; 11 Yan’an Hospital (affiliated with Kunming Medical University), Yunnan, China

**Keywords:** hand, foot, and mouth disease, vaccination, enterovirus 71, COVID-19, epidemical trend, HFMD, EV71

## Abstract

**Background:**

Hand, foot, and mouth disease (HFMD) is a highly contagious viral illness. Understanding the long-term trends of HFMD incidence and its epidemic characteristics under the circumstances of the enterovirus 71 (EV71) vaccination program and the outbreak of COVID-19 is crucial for effective disease surveillance and control.

**Objective:**

We aim to give an overview of the trends of HFMD over the past decades and evaluate the impact of the EV71 vaccination program and the COVID-19 pandemic on the epidemic trends of HFMD.

**Methods:**

Using official surveillance data from the Yunnan Province, China, we described long-term incidence trends and severity rates of HFMD as well as the variation of enterovirus proportions among cases. We conducted the autoregressive integrated moving average (ARIMA) of time series analyses to predict monthly incidences based on given subsets. The difference between the actual incidences and their counterfactual predictions was compared using absolute percentage errors (APEs) for periods after the EV71 vaccination program and the COVID-19 pandemic, respectively.

**Results:**

The annual incidence of HFMD fluctuated between 25.62 cases per 100,000 people in 2008 and 221.52 cases per 100,000 people in 2018. The incidence for men ranged from 30 to 250 cases per 100,000 people from 2008 to 2021, which was constantly higher than that for women. The annual incidence for children aged 1 to 2 years old ranged from 54.54 to 630.06 cases per 100,000 people, which was persistently higher than that for other age groups. For monthly incidences, semiannual peaks were observed for each year. All actual monthly incidences of 2014 to 2015 fell within the predicted 95% CI by the ARIMA(1,0,1)(1,1,0)[12] model. The average APE was 19% for a 2-year prediction. After the EV71 vaccination program, the actual monthly incidence of HFMD was consistently lower than the counterfactual predictions by ARIMA(1,0,1)(1,1,0)[12], with negative APEs ranging from −11% to −229% from January 2017 to April 2018. In the meantime, the proportion of EV71 among the enteroviruses causing HFMD decreased significantly, and the proportion was highly correlated (*r*=0.73, *P*=.004) with the severity rate. After the onset of the COVID-19 pandemic in 2020, the actual monthly incidence of HFMD consistently maintained a lower magnitude compared to the counterfactual predictions—ARIMA(1,0,1)(0,1,0)[12]—from February to September 2020, with considerable negative APEs (ranging from −31% to −2248%).

**Conclusions:**

EV71 vaccination alleviated severe HFMD cases and altered epidemiological trends. The HFMD may also benefit from nonpharmaceutical interventions during outbreaks such as the COVID-19 pandemic. Further development of a multivalent virus vaccine is crucial for effectively controlling HFMD outbreaks. Policymakers should implement nonpharmaceutical interventions and emphasize personal hygiene for routine prevention when appropriate.

## Introduction

Hand, foot, and mouth disease (HFMD) is a viral illness commonly seen in children under 5 years old, characterized by typical manifestations such as oral herpes and rashes on the hands and feet [1]. The incidence of HFMD has been noted in China since 2007, with multiple severe outbreaks. This contagious disease was officially integrated into the National Notifiable Disease Reporting System in 2008 [2]. Laboratory surveillance results showed that the causes of HFMD in China were enterovirus 71 (EV71) and coxsackievirus A16 (CV-A16) in most cases, and EV71 was the most frequently identified serotype among both severe and fatal cases [3]. The first inactivated EV71 whole virus vaccine for preventing severe HFMD was approved by the China Food and Drug Administration in December 2015 [2]. Most regions in China implemented the vaccine in the second half of 2016, and changes in the epidemiology of HFMD have been noted ever since [4].

The changes in epidemiological characteristics were observed in many infectious diseases following the COVID-19 outbreak in China [5]. Under the policy of nonpharmaceutical interventions (NPIs) [6], which includes travel restrictions, contact reductions, and social distancing, diseases caused by respiratory pathogens were found to be particularly impacted by NPIs [7]. Other infectious diseases with alternative transmission modes, such as HFMD, were also influenced by the interventions [8]. Shen et al [9] explored the influence of the COVID-19 pandemic in Xi’an, using the number of HFMD cases per month from 2013 to 2019 as training datasets, and compared the actual number of cases per month with the predictions in 2020 to 2021 generated by a Bayesian structural time series model. A significant decrease (−94.52%) in the number of HFMD cases in the first half-year of 2020 was observed. Another study focusing on the effects of different NPI levels on HFMD in Guangzhou, using cases from 2015 to 2021, elaborated a significant benefit of strong NPIs on HFMD case reduction [10]. Although the implementation of the EV71 vaccination and the COVID-19 pandemic are both potential factors that could alter the epidemiological trend of HFMD, previous investigations have predominantly focused on evaluating the impact of either one of the two factors [9-12]. Therefore, it is essential to conduct quantitative evaluations of both the vaccine and the COVID-19 pandemic on the prevailing trends of HFMD epidemiology.

Yunnan, situated in the southwestern periphery of China, covers an approximate area of 394,000 km^2^, with approximately 47 million inhabitants in 2021 [13]. It plays a pivotal role because of its unique location within the Greater Mekong Subregion, which is an assemblage encompassing sovereign entities including the People’s Republic of China, Cambodia, the Lao People’s Democratic Republic, Myanmar, Thailand, and Vietnam [14]. Collaborations among these regions are closely tied, including public health decisions [15]. Therefore, exploring the epidemiological characteristics of major infectious diseases represented by HFMD in the Yunnan Province is crucial for global health.

This study aims to depict the long-term epidemiological trend of HFMD after its outbreak in the Yunnan Province, China, with quantitative evaluations of the impact of both the EV71 vaccination and the COVID-19 pandemic, using a counterfactual time series modeling approach.

## Methods

### Data Sources

Monthly HFMD surveillance data and demographic statistics from January 1, 2008, to December 31, 2021, in Yunnan were obtained from the National Notifiable Disease Reporting System established by the Chinese Center for Disease Control and Prevention [16]. Most HFMD cases identified in the system were clinically diagnosed [17] and were reported to the system within 24 hours after diagnosis. The criteria for severe cases include those with symptoms such as nervous system involvement; respiratory and circulatory dysfunction; abnormal laboratory test results, implicating increased peripheral blood leukocytes; abnormal cerebrospinal fluid; elevated blood sugar levels; and abnormalities in electroencephalogram, brain and spinal cord magnetic resonance imaging, chest x-ray, and echocardiography findings [18]. Sampling is required for all severe and fatal cases. If no severe case occurs in the county, a random pathogen test of at least 5 cases per month is required. If there are fewer than 5 cases during that month, all cases should be sampled for a pathogen test. Age, gender, and date of illness onset for each case were obtained from the system.

### Ethical Considerations

This study was approved by the Research Ethics Committee of the Yunnan Center for Disease Control and Prevention (2023‐19). The research ethics committee waived informed consent because the study did not involve identifiable personal information.

### Descriptive Analysis

We calculated annual HFMD incidences from 2008 to 2021 and incidences by different groups of gender (male and female) and age (<1, 1 to <2, 2 to <5, 5 to <10, 10 to <15, and ≥15 years). The severity rate was calculated as the number of severe and fatal cases divided by the total number of cases per year. The proportions of HFMD enterovirus subtypes (ie, CV-A16, EV71, and other enteroviruses) per year for the sampled cases were also calculated. The association between the severity rate and the proportion of EV71 among the enterovirus subtypes was investigated using the Spearman correlation analysis.

### Autoregressive Integrated Moving Average Modeling and Counterfactual Analysis

Autoregressive integrated moving average (ARIMA) models (p,d,q)(P,D,Q)s were constructed to predict the subsequent monthly HFMD incidences based on given incidences [19]. The parameters p and P were the autoregressive term and seasonal autoregressive term, respectively; d and D were the respective nonseasonal difference and seasonal difference terms; q and Q were the moving average term and seasonal moving average term, respectively; and s was the periodic term. The model predicted the HFMD incidence despite the EV71 vaccination program and the occurrence of the COVID-19 pandemic, which was also called the counterfactual model. The counterfactual model predicted the incidence and analyzed how the actual incidence diverged from the predictions [20]. Specifically, using the *auto.arima ()* function from the forecast package in R [21], the ARIMA model was built based on the training set, and an optimal set of parameters was automatically chosen by comparing the combinatorial spectrum of parameters according to the rule of the minimum Akaike information criterion or Bayesian information criterion. A Ljung–Box test was performed to check whether the residual sequence was a white noise sequence (*P*>.05 if the residual sequence was a white noise sequence) [22]. The absolute percent error (APE) was calculated based on the actual and predicted monthly incidence ([actual incidence−predicted incidence]/actual incidence).

Several subsets of time series data were used for different purposes: (1) to test the predicting ability of the counterfactual models, monthly incidences of HFMD from January 2008 to December 2013 were used as the training set. The predicted incidence and the actual one from January 2014 to December 2015 were compared using APE. (2) To evaluate the influence of the EV71 vaccination program, monthly incidences of HFMD from January 2008 to December 2016 were used as the training set, and actual incidences from January 2017 to December 2018 were used for comparison. The training set was selected because the vaccination program did not start until late 2016 due to practical issues. (3) To evaluate the influence of the COVID-19 pandemic, monthly incidences from January 2017 to December 2019 were used as the training set, and incidences from January 2020 to December 2021 were compared with its counterpart. The selected ARIMA models for each training set are shown in Table S1 in Multimedia Appendix 1.

The data were analyzed using R, version 4.2.3.

## Results

### Overview

A total of 891,961 HFMD cases were reported in the Yunnan Province, China, from January 1, 2008, to December 31, 2021. Men accounted for 58.13% (n=518,513) of the reported cases. The proportions of cases of children aged <1, 1 to <2, 2 to <5, 5 to <10, 10 to <15, and ≥15 years old were 8.99% (n=80,188), 29.94% (n=267,702), 49.31% (n=439,798), 10.27% (n=91,583), 1.09% (n=9761), and 0.40% (n=3557), respectively. Out of all cases, 10,574 severe and 150 fatal cases were identified. The pathogen test from the samples (n=77,660) showed the proportions of CV-A16, EV71, and other kinds of enteroviruses were 31.96% (n=24,822), 27.72% (n=21,525), and 40.32% (n=31,313), respectively.

### Epidemiological Trends of HFMD

**Figure 1.**
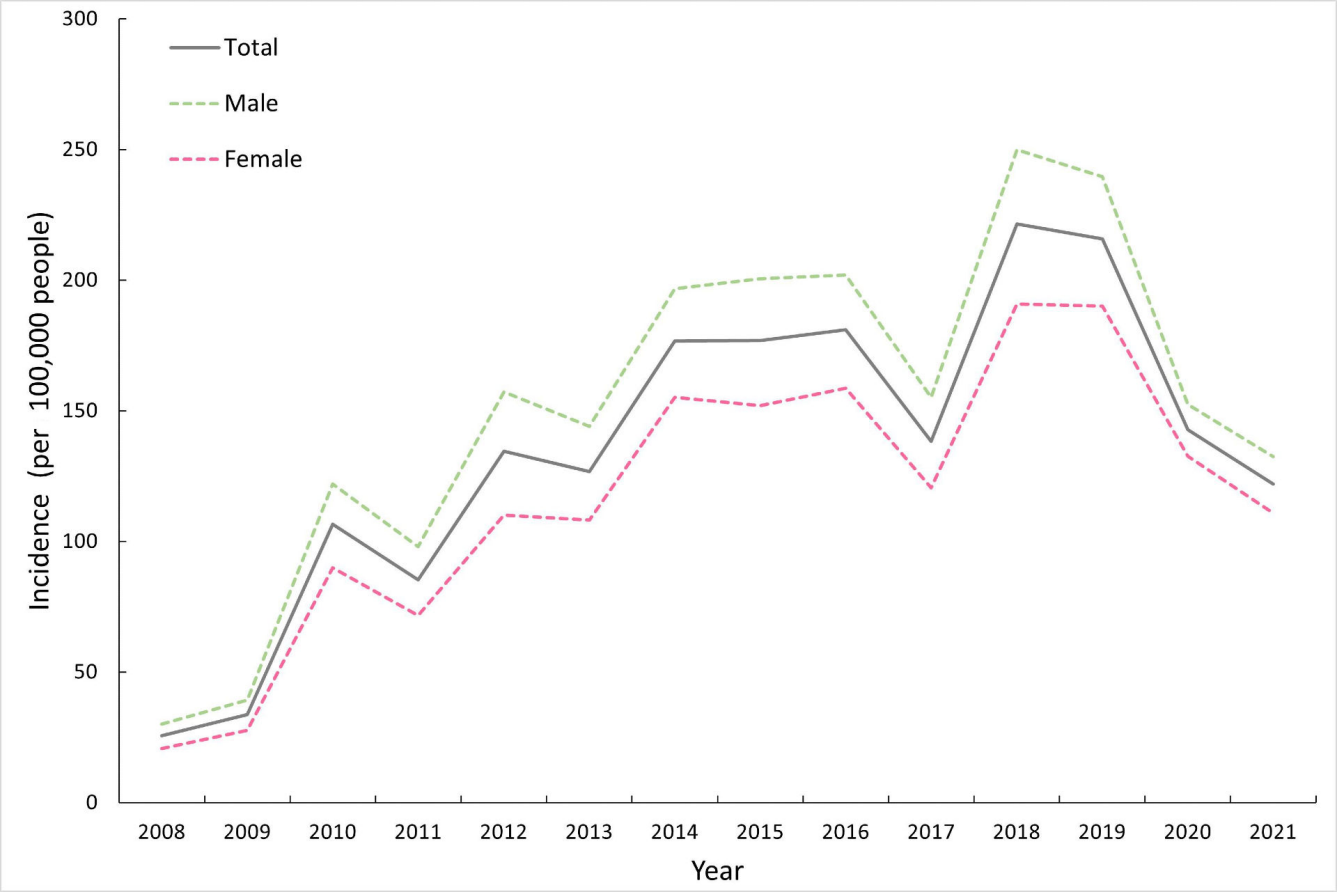
Annual incidences of hand, foot, and mouth disease (per 100,000 people) by sex in the Yunnan Province, China, 2008 to 2021.

**Figure 2.**
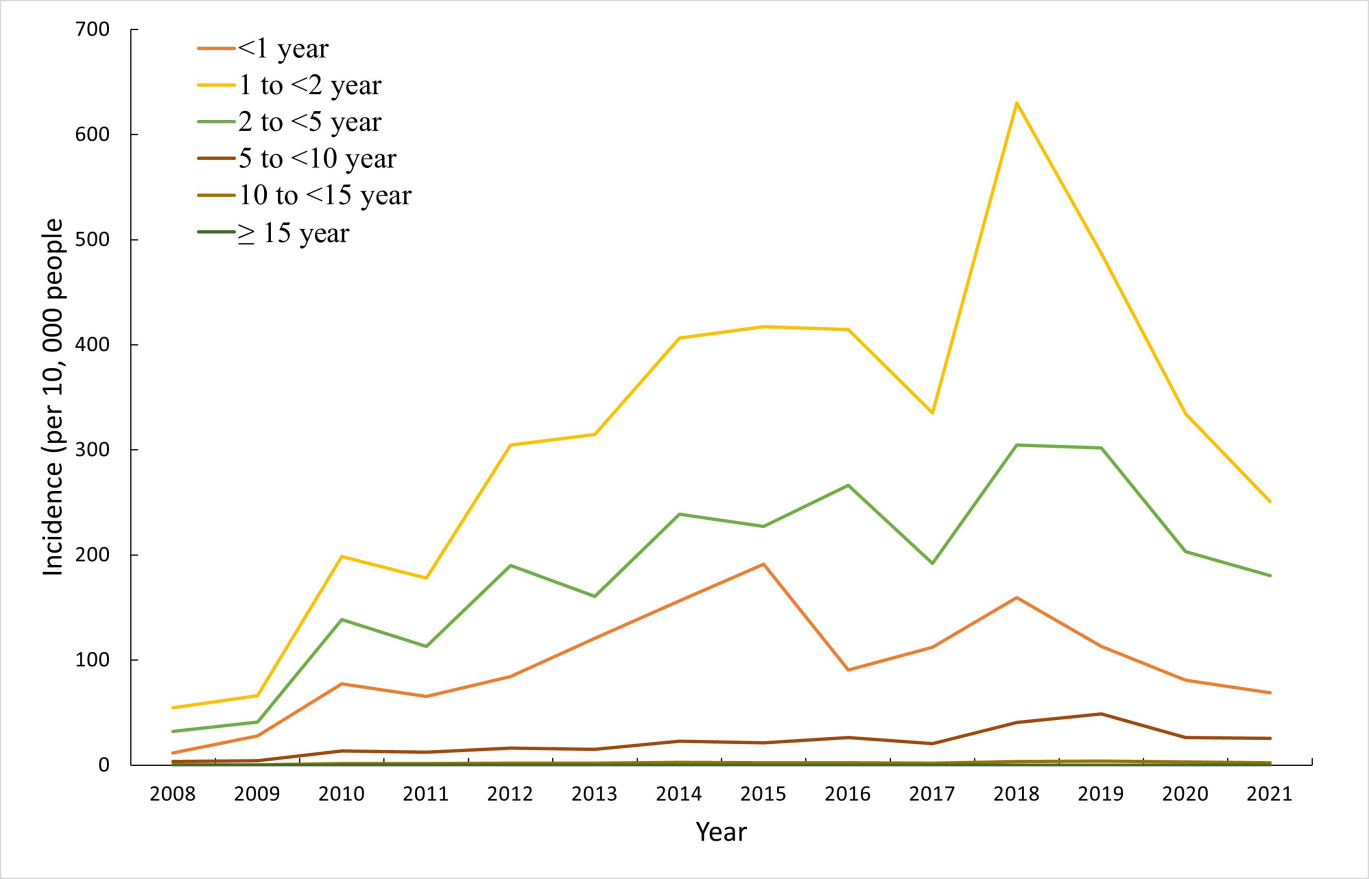
Annual incidences of hand, foot, and mouth disease (per 10,000 people) by age group (in years) in the Yunnan Province, China, 2008 to 2021.

The annual incidence fluctuated between 25.62 cases per 100,000 people in 2008 and 221.52 cases per 100,000 people in 2018. The incidence for men ranged from 30.06 to 249.89 cases per 100,000 people, which were constantly higher than that of women (20.80-190.92 cases per 100,000 people) from 2008 to 2021 (Figure 1). Although children between 2 and 5 years old accounted for more than half of the cases identified, the annual incidence for children aged 1 to 2 years old, ranging from 54.54 to 630.06 cases per 100,000 people, was persistently higher than that for other age groups (Figure 2).

The monthly incidence of HFMD was characterized by 2 peaks each year, showing semiannual peaks (Figure S1 in Multimedia Appendix 1). The peaks included a major one around May, followed by a smaller one in autumn, except for 2020, during which only 1 peak was seen. Notable decreases in incidence were seen in 2017 and 2020. However, an increase was seen in 2018 to 2019.

**Figure 3.**
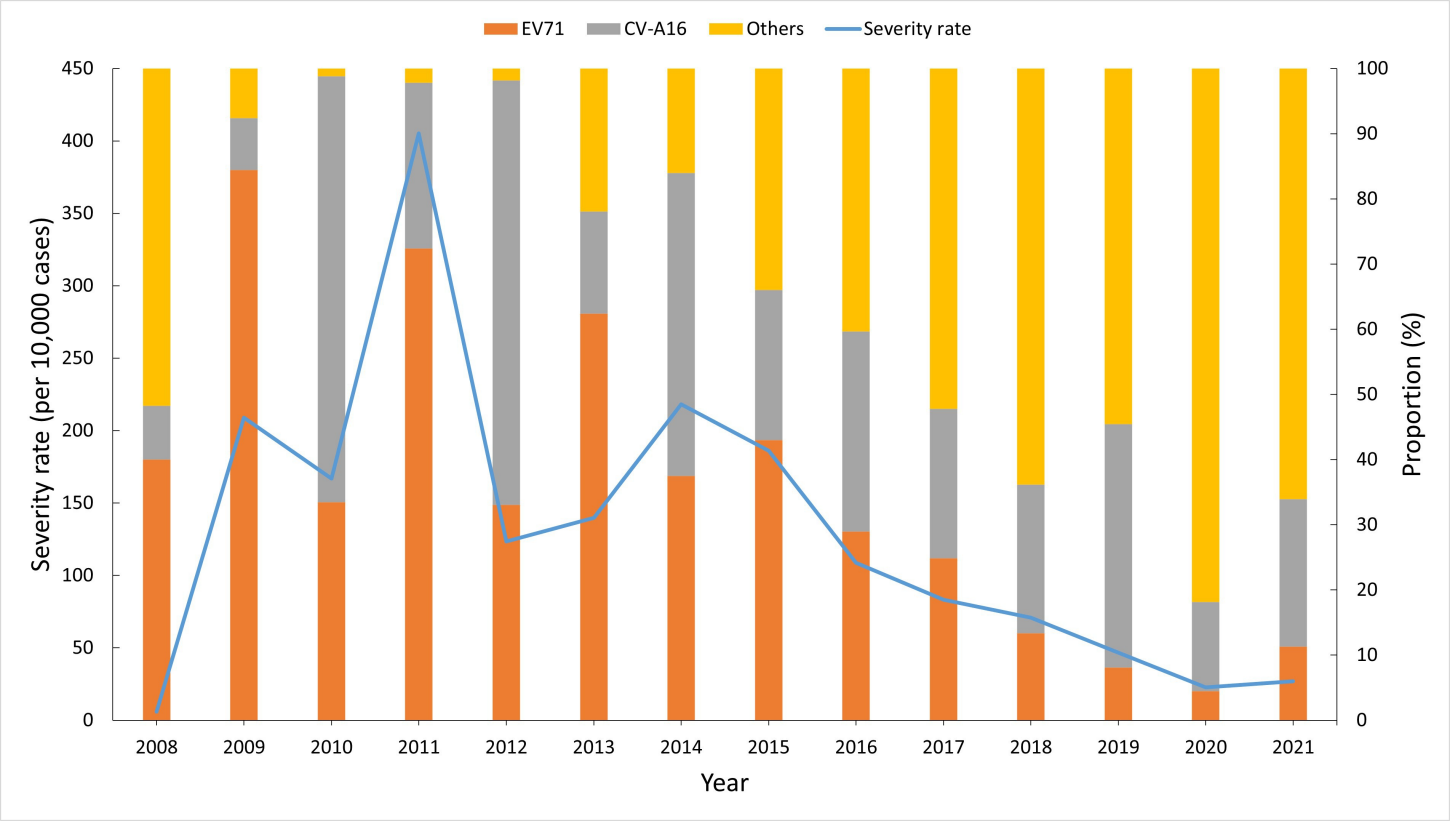
Annual severity rates (per 10,000 cases) of reported hand, foot, and mouth disease patients and proportions of enterovirus subtypes in the Yunnan Province, China, 2008 to 2021.

The severity rate and the proportion of enterovirus subtypes varied over the years (Figure 3). The highest severity rate appeared in 2011 at 405.11 per 10,000 cases, and a notable decline was observed in 2016, with a continuous decrease till the lowest point was reached in 2020 at 22.76 per 10,000 cases. The proportion of EV71 and CV-A16 among all tested enterovirus increased substantially from 2009 to 2012, reaching a peak of 98.18% (5985/6096) in 2012. During this period, the highest proportion of EV71 was found in 2009, accounting for 84% (244/289) of tested cases. On the other hand, the proportion of other enteroviruses generally decreased and remained relatively low during 2009‐2012. The proportion of EV71 and CV-A16 accounted for the major enteroviruses of the tested samples from 2013 to 2016. A turning point was seen in 2017, with a significant increase in the proportion of other types of enteroviruses, accounting for more than half of the tested cases. A continuing decline in the proportion of EV71 was observed till 2020, with it only accounting for 4.49% (279/6210) of cases in that year. In 2020 and 2021, the majority of enteroviruses were other enterovirus subtypes (excluding EV71 and CV-A16), representing 81.82% (5081/6,210) and 66.06% (3972/6013), respectively. Annual severity rates and the proportions of EV71 were highly correlated with a Spearman correlation coefficient of 0.73 (*P*=.004).

### ARIMA Modeling and Counterfactual Analysis

#### Ability of Prediction

**Figure 4.**
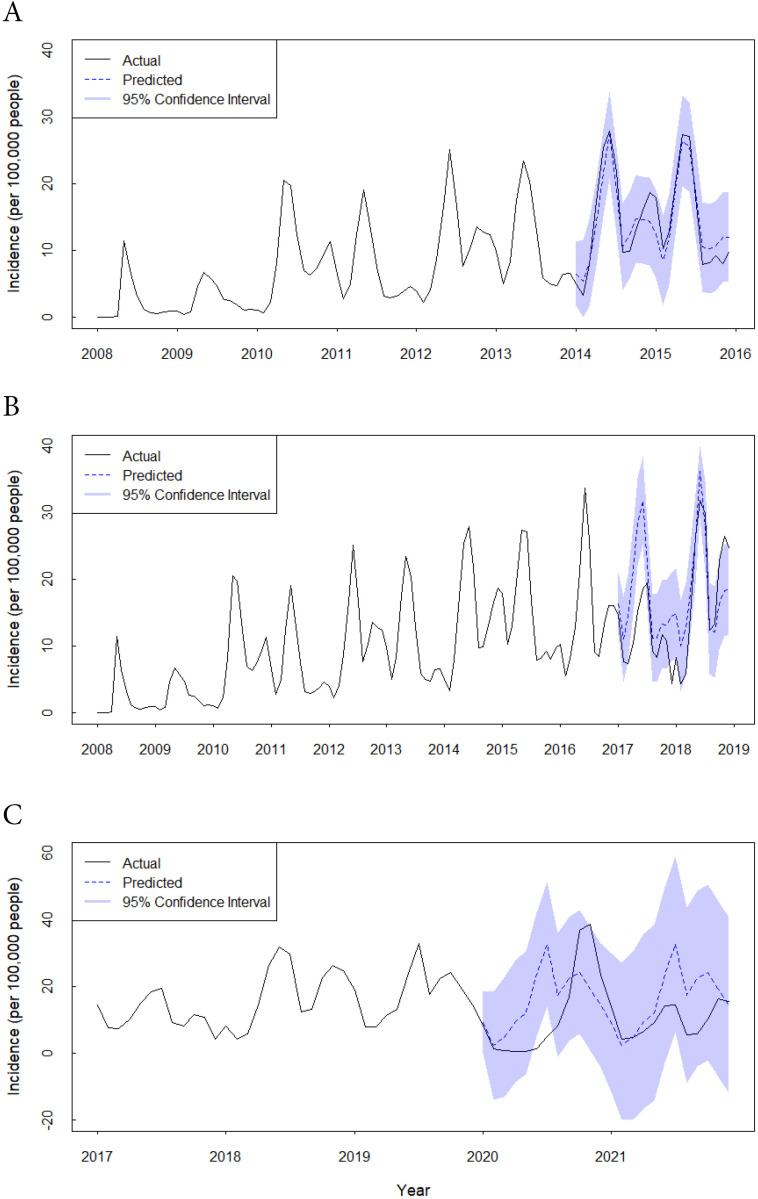
Actual monthly incidences (per 100,000 people) of hand, foot, and mouth disease and the predictions by autoregressive integrated moving average analysis in the Yunnan Province, China, 2008 to 2021.

Using actual incidences from 2008 to 2013 as the training set, the ARIMA(1,0,1)(1,1,0)[12] model predicted the incidences and 95% CIs from January 2014 to December 2015, which are shown in Figure 4A. All actual monthly incidences of 2014 to 2015 fell within the predicted 95% CI (Table S2 in Multimedia Appendix 1). The average APE was 19% for a 2-year prediction.

#### Influence of the EV71 Vaccination Program

Using actual incidences from 2008 to 2016 as the training set, the ARIMA(1,0,1)(1,1,0)[12] model predicted incidences of HFMD for 2017 to 2018, which are are shown in Figure 4B and Table 1. The actual HFMD incidences were continuously lower than the predicted counterparts from January 2017 to April 2018. APEs exceeding –100% were identified during this period, indicating that the actual incidence was less than half of the predicted incidence. The phenomenon was more obvious in April 2017 (APE −107%), December 2017 (APE −229%), February 2018 (APE −127%), and March 2018 (APE −118%). However, an upward trend of the actual incidences was observed from May 2018 onward, surpassing the levels predicted by the model, and fluctuations in monthly incidence resurfaced subsequently. In the end, the actual incidence of HFMD reached nearly 1.5 times higher that of the predicted ones from October to December 2018.

**Table 1.** Actual monthly incidences (per 100,000 people) of hand, foot, and mouth disease and the predictions by autoregressive integrated moving average analysis in the Yunnan Province, China, after the EV71 vaccination program in 2017‐2019.

Month	Actual incidence	Predicted incidence	95% CI	Absolute percentage error, %
Jan 2017	14.71	16.38	(11.61 to 21.14)	−11
Feb 2017	7.73	11.06	(4.70 to 17.42)	−43
Mar 2017	7.40	14.20	(7.64 to 20.76)	−92
Apr 2017	10.39	21.54	(14.95 to 28.13)	−107
May 2017	15.18	28.64	(22.05 to 35.24)	−89
Jun 2017	18.62	31.93	(25.33 to 38.52)	−71
Jul 2017	19.55	22.24	(15.65 to 28.84)	−14
Aug 2017	9.30	11.21	(4.61 to 17.81)	−21
Sep 2017	8.39	11.21	(4.62 to 17.81)	−34
Oct 2017	11.80	13.31	(6.71 to 19.90)	−13
Nov 2017	10.86	13.27	(6.67 to 19.87)	−22
Dec 2017	4.40	14.46	(7.87 to 21.06)	−229
Jan 2018	8.39	14.95	(8.22 to 21.67)	−78
Feb 2018	4.42	10.02	(3.20 to 16.84)	−127
Mar 2018	5.84	12.70	(5.87 to 19.53)	−117
Apr 2018	14.66	18.18	(11.34 to 25.01)	−24
May 2018	26.47	26.13	(19.29 to 32.96)	1
Jun 2018	31.90	36.24	(29.40 to 43.07)	−14
Jul 2018	29.93	27.93	(21.09 to 34.77)	7
Aug 2018	12.43	12.65	(5.82 to 19.49)	−2
Sep 2018	13.35	12.13	(5.29 to 18.96)	9
Oct 2018	22.84	16.31	(9.48 to 23.15)	29
Nov 2018	26.49	18.32	(11.48 to 25.15)	31
Dec 2018	24.80	18.65	(11.82 to 25.49)	25

#### Influence of the COVID-19 Pandemic

Concerning the influence of the COVID-19 pandemic, monthly incidences of HFMD from 2017 to 2019 were used as the training set. Comparisons were made between the actual monthly incidences of 2020‐2021 and the predicted values by the selected ARIMA(1,0,1)(0,1,0)[12] model (Figure 4C and Table 2). After the outbreak of COVID-19, the expected semiannual peaks of monthly incidences disappeared, evidenced by a singular peak occurring later in 2020. In addition, the actual incidence maintained a substantially diminished level compared with the prediction from February to September 2020. The actual HFMD incidences were 0.74, 0.52, and 1.33 per 100,000 people for April, May, and June 2020, respectively, whereas the corresponding predictions were 9.71, 12.21, and 23.57 per 100,000 people, respectively. More than tenfold discrepancies were observed between the actual incidences and the predictions for April, May, and June 2020 (APEs −1212%, −2248%, −1672%, respectively). The actual incidences increased following September 2020, and the semiannual peaks of monthly incidences re-emerged in 2021.

**Table 2.** Actual monthly incidences (per 100,000 people) of hand, foot, and mouth disease and the predictions by autoregressive integrated moving average analysis in the Yunnan Province, China, after the COVID-19 outbreak in 2020‐2021.

Month	Actual incidence	Predicted incidence	95% CI	Absolute percentage error, %
Jan 2020	8.52	9.42	(0.23 to18.61)	11
Feb 2020	1.36	2.44	(−13.73 to 18.61)	−79
Mar 2020	0.85	4.87	(−12.98 to 22.73)	−473
Apr 2020	0.74	9.71	(−8.66 to 28.07)	−1212
May 2020	0.52	12.21	(−6.31 to 30.74)	−2248
Jun 2020	1.33	23.57	(4.99 to 42.15)	−1672
Jul 2020	4.99	32.77	(14.17 to 51.37)	−557
Aug 2020	8.21	17.58	(−1.02 to 36.19)	−114
Sep 2020	17.05	22.40	(3.80 to 41.01)	−31
Oct 2020	36.94	24.37	(5.77 to 42.97)	34
Nov 2020	38.83	19.38	(0.78 to 37.98)	50
Dec 2020	23.52	14.50	(−4.10 to 33.11)	38
Jan 2021	14.11	9.41	(−11.35 to 30.17)	33
Feb 2021	4.40	2.44	(−22.23 to 27.10)	45
Mar 2021	4.82	4.87	(−20.93 to 30.67)	−1
Apr 2021	6.66	9.70	(−16.46 to 35.87)	−46
May 2021	9.32	12.21	(−14.06 to 38.49)	−31
Jun 2021	14.43	23.57	(−2.74 to 49.89)	−63
Jul 2021	14.60	32.77	(6.44 to 59.09)	−124
Aug 2021	5.56	17.58	(−8.75 to 43.91)	−216
Sep 2021	5.77	22.40	(−3.93 to 48.73)	−288
Oct 2021	10.26	24.37	(−1.96 to 50.70)	−138
Nov 2021	16.50	19.38	(−6.95 to 45.71)	−17
Dec 2021	15.59	14.50	(−11.83 to 40.84)	7

## Discussion

### Principle Findings

With in-depth epidemiological and time series analyses of HFMD in the Yunnan Province, we depicted long-term epidemiological trends from 2008 to 2021, with semiannual peaks in its incidence and a notable decrease after the implementation of the EV71 vaccination program in 2017 and the outbreak of COVID-19 in 2020. Moreover, the vaccination campaign reduced the number of severe HFMD cases and shifted the pathogen composition of HFMD. The counterfactual model predicted the incidence without considering the vaccination program and the COVID-19 pandemic, indicating that the vaccination program had alleviated HFMD incidence and that the NPIs during the COVID-19 pandemic may have had a significant beneficial effect on mitigating HFMD.

The annual incidence rate ranged from 25.79 to 653.26 cases per 100,000 people in most Chinese provinces from 2015 to 2019 [23]. The incidence in the Yunnan Province fluctuated between 25.62 and 221.52 cases per 100,000 people, representing a moderate HFMD incidence level across China. We observed a characteristic semiannual pattern in HFMD incidence, with major peaks occurring around May and smaller peaks in autumn. The observed semiannual pattern was also seen in other provinces, like Jiangsu [24]. However, the incidence of HFMD in 2017 and 2020 deviated from this pattern. A significant decrease was seen in early 2017 and 2020, indicating the immediate benefits of vaccination and NPIs in mitigating HFMD transmission.

Similar epidemiological trends regarding the pathogen composition of HFMD were also found in other regions [4]. For example, the number of cases associated with EV71 in the Guangxi Province also decreased significantly following 2017. Cases that tested positive for EV71 comprised 28% of all cases in Guangxi from 2013 to 2015, while this percentage decreased to 13.3% in 2017 to 2019. However, EV71 is still the most prevalent serotype in unvaccinated areas, such as Vietnam and Korea [25,26]. The initial application of the EV71 vaccination program resulted in a reduction in incidence; however, a subsequent resurgence of HFMD incidence was observed in this study. This phenomenon can be attributed to the vaccine’s specific targeting of EV71, which was predominant in the earlier years, leading to a decline in overall incidence. However, as the vaccine induced changes in the pathogen composition of HFMD, its effectiveness in controlling the incidence of other nontargeted enterovirus subtypes was limited, resulting in the observed resurgence in incidence. Nonetheless, the vaccination program did demonstrate significant efficacy in reducing the severity rate among HFMD cases. Although the EV71 vaccination effectively reduced the severity rate and incidence in the early stage, its capacity to control the incidence diminished over time. Thus, a more comprehensive vaccine targeting a broader spectrum of pathogens warrants consideration.

In the early stages of the COVID-19 outbreak, the Chinese government implemented stringent NPIs, including contact reductions, social distancing, and the closure of public spaces like schools [27]. In response to these interventions, the typical semiannual peaks of HFMD incidences shifted into 1 peak per year in 2020, maintaining an extremely low level in Yunnan. The biggest difference between the actual and the predicted incidence of the ARIMA model occurred in early 2020 when the strictest lockdown was mandated by the Chinese government. The most extreme APE of –2248% was observed in May 2020. Previous reports indicated that the incidences of respiratory-transmitted diseases, vector-borne diseases, and other gastrointestinal infectious diseases decreased rapidly during the COVID-19 pandemic in China, especially during the “first-level response” phase in 2020 [28,29]. However, with the implementation of the “normalized control” and “dynamic COVID-zero” measures of less strict contact control in populations [30,31], a resurgence in the incidence of these diseases was observed. This parallels the long-term trends of HFMD observed in our study. Following the reopening of primary schools in September 2020, the incidence rate of HFMD experienced a swift rebound to the single peak in the year, before returning to the pattern similar to previous years.

This study provides valuable insights into the long-term patterns of HFMD incidence and severity rate in the Yunnan Province. By utilizing official surveillance data and applying an ARIMA analysis, this study quantitively assessed the impact of the EV71 vaccination program and the COVID-19 pandemic on the HFMD epidemic. Our study is enhanced by the counterfactual model, which allows for a comprehensive evaluation of the long-term incidence trend of HFMD and provides us with reliable predictions. Moreover, a broad time span and the consideration of 2 possible impactors sets this study apart from previous investigations.

### Limitations

Some limitations of this study should be acknowledged. First, this study assessed the impact of the COVID-19 pandemic on HFMD prevalence regardless of the different levels of NPIs. The influence of other public health interventions beyond vaccination and NPIs during the COVID-19 pandemic was not explicitly examined, potentially confounding the observed associations. Moreover, the ARIMA model, though useful for forecasting HFMD incidence, might not account for all potential confounding factors affecting disease dynamics.

### Conclusion

In conclusion, this is the first study to describe the long-term epidemic trend of HFMD in the Yunnan Province. We quantitatively explored the impact of the EV71 vaccination program and the outbreak of COVID-19 on the epidemic trends of HFMD. The vaccination program effectively reduced severe HFMD cases and altered the composition of enterovirus subtypes. NPIs during the COVID-19 pandemic showed potential benefits in mitigating HFMD transmission. Public health authorities may consider the combined implementation of multivirus vaccination programs and appropriate NPIs to control HFMD outbreaks.

## Supplementary material

10.2196/63146 Multimedia Appendix 1 Supplementary files.

## Abbreviations:

APE: absolute percentage error
ARIMA: autoregressive integrated moving average
CV-A16: coxsackievirus A16
EV71: enterovirus 71
HFMD: hand, foot, and mouth disease
NPI: nonpharmaceutical intervention
