# Supplementary material for: Influence of the Enterovirus 71 Vaccine and the COVID-19 Pandemic on Hand, Foot, and Mouth Disease in China Based on Counterfactual Models: Observational Study
Source: JMIR Public Health Surveill. 2024 Dec 17;10:e63146. doi: 10.2196/63146 (PMC11683655; doi:10.2196/63146)
Supplement: Multimedia Appendix 1 [file publichealth-v10-e63146-s001.docx]

Supplementary Files

Supplementary Table S1. Information of selected models.

| Training set | Selected Model | RMSE | AIC | BIC | Ljung-Box test |
| --- | --- | --- | --- | --- | --- |
| 2008-2013 | ARIMA(1,0,1)(1,1,0)[12] | 2.14 | 293.19 | 303.66 | *P* = 0.9997 |
| 2008-2016 | ARIMA(1,0,1)(1,1,0)[12] | 2.24 | 458.77 | 471.59 | *P* = 0.9992 |
| 2017-2019 | ARIMA(1,0,1)(0,1,0)[12] | 3.66 | 148.87 | 152.41 | *P* = 0.9877 |

Notes: RMSE: Root Mean Square Error. AIC: Akaike’s information criterion, BIC: Bayesian information criterion. Model ARIMA(1,0,1)(1,1,0)[12] is for the verification of the prediction ability of the ARIMA model. Model ARIMA(1,0,1)(1,1,0)[12] is for the comparison of actual monthly incidence and the predictions after the vaccination program. Model ARIMA(1,0,1)(0,1,0)[12] is for the comparison of actual monthly incidence and the prediction after the outbreak of COVID-19.

Supplementary Table S2. Actual and predicted incidence (per 100,000 population) of HFMD in Yunnan Province, China, 2014-2015.

| Month | Actual incidence | Predicted incidence | 95% Confidence Interval | APE (%) |
| --- | --- | --- | --- | --- |
| Jan 2014 | 5.02 | 6.56 | (1.79, 11.32) | -30% |
| Feb 2014 | 3.34 | 5.39 | (-0.84, 11.62) | -61% |
| Mar 2014 | 7.97 | 8.17 | (1.69, 14.65) | -2% |
| Apr 2014 | 17.46 | 14.17 | (7.65, 20.70) | 19% |
| May 2014 | 25.42 | 21.69 | (15.16, 28.22) | 15% |
| Jun 2014 | 27.97 | 27.38 | (20.85, 33.92) | 2% |
| Jul 2014 | 21.94 | 19.08 | (12.55, 25.61) | 13% |
| Aug 2014 | 9.78 | 10.64 | (4.10, 17.17) | -9% |
| Sep 2014 | 9.98 | 12.37 | (5.83, 18.90) | -24% |
| Oct 2014 | 13.03 | 14.79 | (8.26, 21.33) | -14% |
| Nov 2014 | 16.04 | 14.62 | (8.09, 21.16) | 9% |
| Dec 2014 | 18.75 | 14.40 | (7.86, 20.93) | 23% |
| Jan 2015 | 18.00 | 12.50 | (5.86, 19.15) | 31% |
| Feb 2015 | 10.37 | 8.59 | (1.86, 15.31) | 17% |
| Mar 2015 | 12.94 | 11.72 | (4.98, 18.46) | 9% |
| Apr 2015 | 20.91 | 20.06 | (13.32, 26.80) | 4% |
| May 2015 | 27.47 | 26.45 | (19.71, 33.19) | 4% |
| Jun 2015 | 27.20 | 25.61 | (18.86, 32.35) | 6% |
| Jul 2015 | 16.80 | 17.91 | (11.17, 24.65) | -7% |
| Aug 2015 | 7.93 | 10.59 | (3.85, 17.33) | -33% |
| Sep 2015 | 8.20 | 10.33 | (3.59, 17.07) | -26% |
| Oct 2015 | 9.23 | 10.73 | (3.99, 17.47) | -16% |
| Nov 2015 | 8.14 | 12.02 | (5.28, 18.76) | -48% |
| Dec 2015 | 9.78 | 12.02 | (5.28, 18.77) | -23% |

Note: APE: Absolute Percent Error.

Supplementary Figure S1. Monthly incidence of HFMD in Yunnan Province, China, 2008-2021.

Supplementary Figure S2. Monthly incidence of severe HFMD in Yunnan Province, China, 2008-2021.
